# Call for Emergency Action to Limit Global Temperature Increases, Restore Biodiversity, and Protect Health: Wealthy nations must do much more, much faster*

**DOI:** 10.1093/ntr/ntab163

**Published:** 2021-09-06

**Authors:** Lukoye Atwoli, Abdullah H Baqui, Thomas Benfield, Raffaella Bosurgi, Fiona Godlee, Stephen Hancocks, Richard Horton, Laurie Laybourn-Langton, Carlos Augusto Monteiro, Ian Norman, Kirsten Patrick, Nigel Praities, Marcel G M Olde Rikkert, Eric J Rubin, Peush Sahni, Richard Smith, Nick Talley, Sue Turale, Damián Vázquez

**Affiliations:** 1 Editor in Chief, East African Medical Journal; 2 Editor in Chief, Journal of Health, Population and Nutrition; 3 Editor in Chief, Danish Medical Journal; 4 Editor in Chief, PLOS Medicine; 5 Editor in Chief, The BMJ; 6 Editor in Chief, British Dental Journal; 7 Editor in Chief, The Lancet; 8 Senior Adviser, UK Health Alliance on Climate Change; 9 Editor in Chief, Revista de Saúde Pública; 10 Editor in Chief, International Journal of Nursing Studies; 11 Interim Editor in Chief, CMAJ; 12 Executive Editor, Pharmaceutical Journal; 13 Editor in Chief, Dutch Journal of Medicine; 14 Editor in Chief, NEJM; 15 Editor in Chief, National Medical Journal of India; 16 Chair, UK Health Alliance on Climate Change; 17 Editor in Chief, Medical Journal of Australia; 18 Editor in Chief, International Nursing Review; 19 Editor in Chief, Pan American Journal of Public Health

The UN General Assembly in September 2021 will bring countries together at a critical time for marshalling collective action to tackle the global environmental crisis. They will meet again at the biodiversity summit in Kunming, China, and the climate conference (COP26) in Glasgow, UK. Ahead of these pivotal meetings, we—the editors of health journals worldwide—call for urgent action to keep average global temperature increases below 1.5°C, halt the destruction of nature, and protect health.

Health is already being harmed by global temperature increases and the destruction of the natural world, a state of affairs health professionals have been bringing attention to for decades.^1^ The science is unequivocal; a global increase of 1.5°C above the pre-industrial average and the continued loss of biodiversity risk catastrophic harm to health that will be impossible to reverse.^2,3^ Despite the world’s necessary preoccupation with COVID-19, we cannot wait for the pandemic to pass to rapidly reduce emissions.

Reflecting the severity of the moment, this editorial appears in health journals across the world. We are united in recognizing that only fundamental and equitable changes to societies will reverse our current trajectory.

The risks to health of increases above 1.5°C are now well established.^2^ Indeed, no temperature rise is “safe”. In the past 20 years, heat-related mortality among people aged over 65 has increased by more than 50%.^4^ Higher temperatures have brought increased dehydration and renal function loss, dermatological malignancies, tropical infections, adverse mental health outcomes, pregnancy complications, allergies, and cardiovascular and pulmonary morbidity and mortality.^5,6^ Harms disproportionately affect the most vulnerable, including among children, older populations, ethnic minorities, poorer communities, and those with underlying health problems.^2,4^

Global heating is also contributing to the decline in global yield potential for major crops, falling by 1.8–5.6% since 1981; this, together with the effects of extreme weather and soil depletion, is hampering efforts to reduce undernutrition.^4^ Thriving ecosystems are essential to human health, and the widespread destruction of nature, including habitats and species, is eroding water and food security and increasing the chance of pandemics.^3,7,8^

The consequences of the environmental crisis fall disproportionately on those countries and communities that have contributed least to the problem and are least able to mitigate the harms. Yet no country, no matter how wealthy, can shield itself from these impacts. Allowing the consequences to fall disproportionately on the most vulnerable will breed more conflict, food insecurity, forced displacement, and zoonotic disease—with severe implications for all countries and communities. As with the COVID-19 pandemic, we are globally as strong as our weakest member.

Rises above 1.5°C increase the chance of reaching tipping points in natural systems that could lock the world into an acutely unstable state. This would critically impair our ability to mitigate harms and to prevent catastrophic, runaway environmental change.^9,10^

## Global Targets Are Not Enough

Encouragingly, many governments, financial institutions, and businesses are setting targets to reach net-zero emissions, including targets for 2030. The cost of renewable energy is dropping rapidly. Many countries are aiming to protect at least 30% of the world’s land and oceans by 2030.^11^

These promises are not enough. Targets are easy to set and hard to achieve. They are yet to be matched with credible short and longer term plans to accelerate cleaner technologies and transform societies. Emissions reduction plans do not adequately incorporate health considerations.^12^ Concern is growing that temperature rises above 1.5°C are beginning to be seen as inevitable, or even acceptable, to powerful members of the global community.^13^ Relatedly, current strategies for reducing emissions to net zero by the middle of the century implausibly assume that the world will acquire great capabilities to remove greenhouse gases from the atmosphere.^14,15^

This insufficient action means that temperature increases are likely to be well in excess of 2°C,^16^ a catastrophic outcome for health and environmental stability. Critically, the destruction of nature does not have parity of esteem with the climate element of the crisis, and every single global target to restore biodiversity loss by 2020 was missed.^17^ This is an overall environmental crisis.^18^

Health professionals are united with environmental scientists, businesses, and many others in rejecting that this outcome is inevitable. More can and must be done now—in Glasgow and Kunming—and in the immediate years that follow. We join health professionals worldwide who have already supported calls for rapid action.^1,19^

Equity must be at the centre of the global response. Contributing a fair share to the global effort means that reduction commitments must account for the cumulative, historical contribution each country has made to emissions, as well as its current emissions and capacity to respond. Wealthier countries will have to cut emissions more quickly, making reductions by 2030 beyond those currently proposed^20,21^ and reaching net-zero emissions before 2050. Similar targets and emergency action are needed for biodiversity loss and the wider destruction of the natural world.

To achieve these targets, governments must make fundamental changes to how our societies and economies are organized and how we live. The current strategy of encouraging markets to swap dirty for cleaner technologies is not enough. Governments must intervene to support the redesign of transport systems, cities, production and distribution of food, markets for financial investments, health systems, and much more. Global coordination is needed to ensure that the rush for cleaner technologies does not come at the cost of more environmental destruction and human exploitation.

Many governments met the threat of the COVID-19 pandemic with unprecedented funding. The environmental crisis demands a similar emergency response. Huge investment will be needed, beyond what is being considered or delivered anywhere in the world. But, such investments will produce huge positive health and economic outcomes. These include high-quality jobs, reduced air pollution, increased physical activity, and improved housing and diet. Better air quality alone would realize health benefits that easily offset the global costs of emissions reductions.^22^

These measures will also improve the social and economic determinants of health, the poor state of which may have made populations more vulnerable to the COVID-19 pandemic.^23^ But, the changes cannot be achieved through a return to damaging austerity policies or the continuation of the large inequalities of wealth and power within and between countries.

## Cooperation Hinges on Wealthy Nations Doing More

In particular, countries that have disproportionately created the environmental crisis must do more to support low- and middle-income countries to build cleaner, healthier, and more resilient societies. High-income countries must meet and go beyond their outstanding commitment to provide $100bn a year, making up for any shortfall in 2020 and increasing contributions to and beyond 2025. Funding must be equally split between mitigation and adaptation, including improving the resilience of health systems.

Financing should be through grants rather than loans, building local capabilities and truly empowering communities, and should come alongside forgiving large debts, which constrain the agency of so many low-income countries. Additional funding must be marshalled to compensate for inevitable loss and damage caused by the consequences of the environmental crisis.

As health professionals, we must do all we can to aid the transition to a sustainable, fairer, resilient, and healthier world. Alongside acting to reduce the harm from the environmental crisis, we should proactively contribute to global prevention of further damage and action on the root causes of the crisis. We must hold global leaders to account and continue to educate others about the health risks of the crisis. We must join in the work to achieve environmentally sustainable health systems before 2040, recognizing that this will mean changing clinical practice. Health institutions have already divested more than $42bn of assets from fossil fuels; others should join them.^4^

The greatest threat to global public health is the continued failure of world leaders to keep the global temperature rise below 1.5°C and to restore nature. Urgent, society-wide changes must be made and will lead to a fairer and healthier world. We, as editors of health journals, call for governments and other leaders to act, marking 2021 as the year that the world finally changes course.

## Declaration of Interests

*We have read and understood BMJ policy on declaration of interests and FG serves on the executive committee for the UK Health Alliance on Climate Change and is a Trustee of the Eden Project. RS is the chair of Patients Know Best, has stock in UnitedHealth Group, has done consultancy work for Oxford Pharmagenesis, and is chair of the Lancet Commission of the Value of Death. None further declared.*

*Provenance and peer review*: Commissioned; not externally peer reviewed.

## References

[1] In support of a health recovery. https://healthyrecovery.net

[2] Intergovernmental Panel on Climate Change. Summary for policymakers. In: Global warming of 1.5°C. An IPCC special report on the impacts of global warming of 1.5°C above pre-industrial levels and related global greenhouse gas emission pathways, in the context of strengthening the global response to the threat of climate change, sustainable development, and efforts to eradicate poverty. 2018. https://www.ipcc.ch/sr15/

[3] Intergovernmental Science-Policy Platform on Biodiversity and Ecosystem Services. Summary for policymakers: the global assessment report on biodiversity and ecosystem services. 2019. https://ipbes.net/sites/default/files/2020-02/ipbes_global_assessment_report_summary_for_policymakers_en.pdf

[4] Watts N, Amann M, Arnell N, et al The 2020 report of the Lancet Countdown on health and climate change: responding to converging crises. Lancet. 2021;397:129–170. 33278353 10.1016/S0140-6736(20)32290-X PMC7616803

[5] Rocque RJ, Beaudoin C, Ndjaboue R, et al Health effects of climate change: an overview of systematic reviews. BMJ Open. 2021;11:e046333. doi:10.1136/bmjopen-2020-046333 PMC8191619 34108165

[6] Haines A, Ebi K. The imperative for climate action to protect health. N Engl J Med 2019;380:263–273. 30650330 10.1056/NEJMra1807873

[7] United Nations Environment Programme and International Livestock Research Institute. *Preventing the next pandemic: zoonotic diseases and how to break the chain of transmission*. 2020. https://72d37324-5089-459c-8f70-271d19427cf2.filesusr.com/ugd/056cf4_b5b2fc067f094dd3b2250cda15c47acd.pdf

[8] IPCC. 2019: Summary for policymakers. In: Climate change and land: an IPCC special report on climate change, desertification, land degradation, sustainable land management, food security, and greenhouse gas fluxes in terrestrial ecosystems. Forthcoming.

[9] Lenton TM, Rockström J, Gaffney O, et al Climate tipping points—too risky to bet against. Nature. 2019;575:592–595. 31776487 10.1038/d41586-019-03595-0

[10] Wunderling N, Donges JF, Kurths J, Winkelmann R. Interacting tipping elements increase risk of climate domino effects under global warming. Earth Syst Dyn. 2021;12:601–619.

[11] High Ambition Coalition. https://www.hacfornatureandpeople.org

[12] Global Climate and Health Alliance. Are national climate commitments enough to protect our health? https://climateandhealthalliance.org/initiatives/healthy-ndcs/ndc-scorecards/

[13] Climate strikers: Open letter to EU leaders on why their new climate law is ‘surrender.’ Carbon Brief 2020. https://www.carbonbrief.org/climate-strikers-open-letter-to-eu-leaders-on-why-their-new-climate-law-is-surrender

[14] Fajardy M, Köberle A, MacDowell N, Fantuzzi A. “BECCS deployment: a reality check.” Grantham Institute briefing paper 28, 2019. https://www.imperial.ac.uk/media/imperial-college/grantham-institute/public/publications/briefing-papers/BECCS-deployment---a-reality-check.pdf

[15] Anderson K, Peters G. The trouble with negative emissions. Science. 2016;354:182–183. 27738161 10.1126/science.aah4567

[16] Climate action tracker. https://climateactiontracker.org

[17] Secretariat of the Convention on Biological Diversity. *Global biodiversity outlook 5*. 2020. https://www.cbd.int/gbo5

[18] Steffen W, Richardson K, Rockström J, et al Sustainability. Planetary boundaries: guiding human development on a changing planet. Science 2015;347:1259855. doi:10.1126/science.1259855 25592418

[19] UK Health Alliance. Our calls for action. http://www.ukhealthalliance.org/cop26/

[20] Climate Action Tracker. *Warming projections global update: May 2021*. https://climateactiontracker.org/documents/853/CAT_2021-05-04_Briefing_Global-Update_Climate-Summit-Momentum.pdf

[21] United Nations Environment Programme. Emissions gap report 2020. UNEP, 2020.

[22] Markandya A, Sampedro J, Smith SJ, et al Health co-benefits from air pollution and mitigation costs of the Paris Agreement: a modelling study. Lancet Planet Health. 2018;2:e126–e133. doi:10.1016/S2542-5196(18)30029-9 29615227

[23] Paremoer L, Nandi S, Serag H, Baum F. Covid-19 pandemic and the social determinants of health. BMJ. 2021;372:n129. 33509801 10.1136/bmj.n129 PMC7842257

